# Hypogonadism and Low Testosterone Levels as a Side Effect of Methadone and Buprenorphine

**DOI:** 10.5812/ijhrba.6539

**Published:** 2012-07-25

**Authors:** Ayman Fareed

**Affiliations:** 1 Department of Psychiatry and Behavioral Sciences, Emory University School of Medicine, Atlanta, USA

**Keywords:** Buprenorphine, Metadone, Testis, Histopathology

*Dear Editor*

The article is an animal study which addresses an important clinical issue i.e. hypogonadism and low testosterone levels as a side effect of methadone and buprenorphine (1). This is not an uncommon side effect which we see in patients receiving long term opioid maintenance treatment. Since methadone has been in use since the 1960s, clinicians became aware of this problem and it is usually addressed and treated as needed. On the contrary, buprenorphine has been available for less than a decade. Therefore there is little information available about this possible side effect for patients on buprenorphine. A clinical trial (2) compared methadone to buprenorphine regarding their effect on Erectile Dysfunction (ED) and hormone levels. They found that men on Methadone Maintenance Treatment (MMT), but not Buprenorphine Maintenance Treatment (BMT), had high prevalence of ED, related to hypogonadism and depression. However, this study is limited by the small sample size. Another study (3) showed that patients treated with buprenorphine had a significantly higher testosterone level (P < 0.0001) and a significantly lower frequency of sexual dysfunction (P < 0.0001) compared with patients treated with methadone. Again this study was limited by the small sample size. A case series (4) reported hypogonadism in 10 patients receiving BMT without comparing it to methadone. The pharmacological profile of buprenorphine is different than methadone. Buprenorphine is a partial mu receptor agonist (5) and not a full agonist like methadone. This could be associated with reduced suppression of the central Luteinizing Hormone (LH) compared to methadone and consequently less impact on the testosterone level. Most of the available data did favor buprenorphine over methadone in regard to their effects on hypogonadism by utilizing hormonal levels. The current study has the advantage of using histopathological samples of the rat testicles to confirm the relationship between the hypogonadism and low testosterone level in this population. While this is an animal study, it adds to the knowledge that is currently available. This study may open the door to investigate this side effect at a different level. Future studies need to examine the direct relationship between opioid agonist treatment and testicular atrophy as the possible reason for hypogonadism in this population. There is a need for more studies to investigate this side effect.
